# Thonningianin A ameliorates acetaminophen-induced liver injury by activating GPX4 and modulating endoplasmic reticulum stress

**DOI:** 10.3389/fphar.2025.1531277

**Published:** 2025-02-20

**Authors:** Shanglei Lai, Yingyan Ye, Qinchao Ding, Xiaokai Hu, Ai Fu, Lan Wu, Wenjing Cao, Qingsheng Liu, Xiaobing Dou, Xuchen Qi

**Affiliations:** ^1^ Department of Medical Research Center, Shaoxing People’s Hospital, Shaoxing, Zhejiang, China; ^2^ Hangzhou Medical College Affiliated Lin’an People’s Hospital, The First People’s Hospital of Hangzhou Lin’an District, Hangzhou, Zhejiang, China; ^3^ School of Public Health, Zhejiang Chinese Medical University, Hangzhou, Zhejiang, China; ^4^ School of Life Science, Zhejiang Chinese Medical University, Hangzhou, Zhejiang, China; ^5^ Institute of Hepatology and Epidemiology, Affiliated Hangzhou Xixi Hospital, Zhejiang Chinese Medical University, Hangzhou, Zhejiang, China; ^6^ Hangzhou Hospital of Traditional Chinese Medicine Affiliated to Zhejiang Chinese Medical University, Hangzhou, Zhejiang, China; ^7^ Department of Neurosurgery, Sir Run Run Shaw Hospital, Zhejiang University School of Medicine, Hangzhou, Zhejiang, China; ^8^ Department of Neurosurgery, Shaoxing People’s Hospital, Shaoxing, Zhejiang, China

**Keywords:** thonningianin A, Acetaminophen, acute liver injury, GPx4, endoplasmic reticulum stress, hepatotoxicity

## Abstract

**Introduction:**

Acetaminophen (APAP) is widely used as an analgesic and antipyretic. However overdose APAP can lead to acute liver injury (ALI), representing a significant challenge for public health due to limited treatment options. Current research highlights the need for safer and more effective therapies for APAP-induced liver injury, especially those that target oxidative and endoplasmic reticulum (ER) stress pathways. This study investigates the protective effects of Thonningianin A (TA), a flavonoid compound derived from *Penthorum chinense Pursh*, in mitigating APAP-induced hepatotoxicity.

**Methods:**

The experimental design involved administering TA at doses of 20 mg/kg and 40 mg/kg to C57BL/6 mice prior to inducing hepatotoxicity with APAP.

**Results and discussion:**

TA treatment significantly lowered plasma ALT and AST levels, inhibited the production of inflammatory cytokines, and reduced oxidative stress markers in liver tissues. Furthermore, TA modulated apoptosis-related proteins by increasing BCL-2 expression while decreasing CHOP and BAX levels. It alleviated endoplasmic reticulum (ER) stress by downregulating GRP78, p-PERK, and ATF4. Notably, liver-specific GPX4 knockdown, achieved through AAV-8-mediated shRNA delivery, abolished the hepatoprotective effects of TA, underscoring GPX4’s essential role in mediating TA-induced hepatoprotection. These findings suggest TA as a promising therapeutic agent in managing APAP-induced liver injury, with its unique action on both oxidative and ER stress pathways contributing to its hepatoprotective efficacy.

## 1 Introduction

Acetaminophen (APAP) is a widely utilized analgesic used analgesic and antipyretic agent in clinical practice; however, excessive intake can lead to severe hepatotoxicity, which may result in acute liver failure (Jaeschke et al., 2021; Chilvery et al., 2023). The underlying mechanism of APAP-induced hepatotoxicity is closely related to the formation of N-acetyl-p-benzoquinone imine (NAPQI), a highly reactive metabolite produced through cytochrome P450-mediated biotransformation (Chen et al., 2022; Luyendyk et al., 2024). Normally, NAPQI is detoxified through conjugation with glutathione (GSH). However, in cases of overdose, GSH stores are rapidly depleted, leading to the accumulation of NAPQI. The excessive binding of NAPQI to mitochondrial membrane proteins disrupts the electron transport chain, impairing ATP synthesis and reducing mitochondrial antioxidant defenses. Additionally, NAPQI accumulation increases the production of reactive oxygen species (ROS), which react with nitric oxide to form peroxynitrite. These reactive compounds directly damage mitochondrial DNA, leading to mitochondrial dysfunction and further exacerbating oxidative stress (Du et al., 2016; Cai et al., 2022; Liao et al., 2023). In conclusion, NAPQI can covalently bind to hepatocellular proteins, causing oxidative damage and cell death (Chowdhury et al., 2020).

Glutathione peroxidase 4 (GPX4) is a selenoprotein essential for cellular defense against oxidative stress, with a specific function in reducing lipid peroxides and maintaining redox homeostasis (Nishida Xavier da Silva et al., 2022; Pei et al., 2023; Xie et al., 2023). GPX4 catalyzes the reduction of lipid peroxides to their corresponding alcohols using glutathione as a reducing cofactor. By mitigating lipid peroxidation, GPX4 protects the integrity of cellular membranes and lipoproteins, primarily through the reduction of phospholipid hydroperoxides (PLOOH). Furthermore, GPX4 inhibits arachidonic acid lipoxygenase (ALOX), thereby limiting the propagation of oxidative damage. The enzyme’s antioxidant properties are critical in preventing the accumulation of lipid peroxides and shielding cells from oxidative stress-induced damage (Imai et al., 2017; Maiorino et al., 2018; Ursini and Maiorino, 2020). In contrast, GPX4 inhibition exacerbates lipid peroxidation and oxidative stress, severely compromising hepatocyte integrity. Additionally, GSH depletion exacerbates ROS generation, further aggravating cellular injury (Zhang et al., 2021; Cheng et al., 2022; Tang et al., 2022). Given the central role of oxidative stress in APAP-induced liver damage, ALI poses a serious public health concern, highlighting the need for effective therapeutic strategies aimed at mitigating oxidative stress and preserving hepatocellular function. In addition to oxidative stress, the endoplasmic reticulum (ER) also plays a pivotal role in developing APAP-induced liver injury (Tan et al., 2020; Luo et al., 2024). The ER is responsible for proper protein folding and secretion, maintaining cellular homeostasis. Disruption of ER function leads to the accumulation of misfolded or unfolded proteins, a condition known as ER stress (Wiseman et al., 2022). ER stress has been implicated in various liver diseases and is recognized as a critical contributor to APAP-induced liver injury. Previous studies have demonstrated that the inhibition of ER stress represents a practical therapeutic approach for reducing liver injury (Kusama et al., 2017; Zhang et al., 2022; Ajoolabady et al., 2023).

The search for effective agents to alleviate ER stress and oxidative damage in ALI has recently garnered attention (Guimaraes et al., 2023; Xu et al., 2023; Li Y. et al., 2024), with traditional Chinese medicine (TCM) emerging as a promising source of hepatoprotective compounds. Among these, Penthorum chinense Pursh (PCP), a medicinal and edible plant species, has demonstrated notable hepatoprotective properties. UHPLC-DAD-TOF/MS combined bioassays revealed that Thonningianin A (TA), a primary bioactive flavonoid in PCP (Lu et al., 2012; Zhang et al., 2015; Zhou et al., 2022), has been shown to possess potent anti-inflammatory (Zhou et al., 2022), antioxidant (Sun X. et al., 2020), and antibacterial (Wang et al., 2024) activities. Additionally, TA exerts various beneficial effects on conditions such as diabetic vascular calcification (Shen et al., 2023), diabetic nephropathy (Zhang et al., 2024), and Alzheimer’s disease (Yu et al., 2024).

The present study investigates the hepatoprotective effects of TA against APAP-induced liver injury in mice, focusing on its regulation of oxidative stress and ER stress pathways. Despite previous studies highlighting the involvement of oxidative and ER stress in APAP-induced hepatotoxicity, the precise molecular mechanisms through which therapeutic agents like TA mitigate these effects remain poorly understood. This study aims to address this knowledge gap by elucidating the specific mechanisms through which TA alleviates oxidative damage, modulates ER stress, and preserves hepatocyte integrity. Furthermore, this research evaluates TA’s potential as a novel therapeutic candidate for the prevention and treatment of acute liver injury, thereby contributing to the development of effective interventions for drug-induced hepatotoxicity.

## 2 Materials and methods

### 2.1 Plant material and reagents

Thonningianin A (TA, Cat. No: B21953; Purity ≥ 98%) was purchased from Shanghai Yuanye Biotechnology Co., Ltd. (Shanghai, China). Acetaminophen (APAP, Cat. No: HY-66005) was purchased from MedChemExpress (State of New Jersey, United States). Sodium carboxymethyl cellulose (CMC-Na, Cat. No: GC26239) was purchased from GLPBIO (Montclair, United States).

### 2.2 Animals experiments

Mice of the C57BL/6 strain, at the age of 8 weeks, were procured from Beijing Vital River Laboratory Animal Technology Co., Ltd. These animals were maintained under controlled environmental conditions, with the ambient temperature regulated at 25°C ± 2°C and relative humidity maintained at 55% ± 5%. Additionally, a diurnal lighting regimen of 12 h of light followed by 12 h of darkness was implemented. After an acclimatization period, the mice were randomly assigned to groups based on body weight. Mice with hepatocyte-targeted knockdown of GPX4 were created through the intravenous administration of recombinant adeno-associated viral (AAV) serotype 8 vectors (Xingzheng Biotechnology Co., Ltd., China). These vectors contained a shRNA sequence specific to murine GPX4 under the control of a liver cell-specific promoter. The AAV8-mediated gene transfer was performed via the lateral tail vein 1 week prior to the commencement of experimental procedures. Following a 16-h fast, the mice were orally administered with TA (20 mg/kg or 40 mg/kg, dissolved in 0.5% CMC-Na) (Zhang et al., 2024), the control and APAP groups were administered intragastrically with the same volume of 0.5% CMC-Na. Two hours later, they were intraperitoneally injected with 300 mg/kg of APAP (Du et al., 2020). Specify the volume of physiological saline administered to the control group for consistency. Twelve hours post-injection, the mice were euthanized, and blood and liver samples were collected for further analysis. All experimental procedures involving animals in this study were conducted according to the guidelines approved by the Animal Ethics Committee of Zhejiang Chinese Medical University (approval number: IACUC-20231225-08).

### 2.3 Biochemical analysis

Mice were anesthetized with Zoletil 50 solution [50 mg/kg body weight, intraperitoneally (i.p.)]. Blood samples were obtained through the inferior vena cava done post-euthanasia, subsequent to which the samples underwent centrifugation at a speed of 3,000 rpm for a duration of 10 min. Plasma levels of alanine aminotransferase (ALT) and aspartate aminotransferase (AST) were measured using commercial assay kits (Nanjing Jiancheng Bio Co. Nanjing, China). To assess hepatic biochemical indicators, liver tissue was homogenized using phosphate-buffered saline (PBS). Hepatic levels of Caspase 3, malondialdehyde (MDA), superoxide dismutase (SOD), and catalase (CAT) were determined using respective commercial assay kits (Beyotime Biotechnology, Shanghai, China). Glutathione peroxidase (GSH-Px) activity in the liver was measured using a commercial assay kit (Nanjing Jiancheng Bio Co. Nanjing, China). GSH and GSSG were determined using commercial assay kits (Beyotime Biotechnology, Shanghai, China). The concentrations of Interleukin-1β (IL-1β), Interleukin-6 (IL-6), Tumor necrosis factor-alpha (TNF-α), and Monocyte chemoattractant protein-1 (MCP-1) were quantified utilizing commercial ELISA kits (ELK Biotechnology, Wuhan, China). To perform the ELISA assay, the following steps were meticulously executed: 100 mg of liver samples were homogenized with 1 mL of PBS. The samples were subsequently lysed on ice for 30 min. The lysate was then collected by centrifugation at 5,000 g for 10 min. The supernatant was collected, and 100 μL of it was aliquoted for the determination of protein concentration using the BCA protein assay kit (Beyotime, Shanghai, China). 100 μL of the sample were pipetted into the wells of an ELISA plate and incubated at 37°C for 80 min. The supernatant was then removed, the wells were dried by tapping, and three washes with the washing buffer were performed. Subsequently, 100 μL of biotin-conjugated antibody solution was added to each well, the plate was sealed with an adhesive cover, and incubated at 37°C for 50 min. The supernatant was discarded and the plate was washed three times. Next, 100 μL of enzyme-conjugated secondary antibody solution was added to each well and incubated at 37°C for 50 min. The supernatant was discarded and the plate was washed five times. Then, 90 μL of TMB substrate was added to each well and incubated at 37°C in the dark for 20 min. The enzymatic reaction was terminated by adding 50 μL of stop solution to each well. The OD was immediately measured at 450 nm. In accordance with the manufacturer’s instructions for the ELISA kits utilized, the inter-assay and intra-assay coefficients of variation (CV%) for IL-1β, IL-6, TNF-α, and MCP-1 were maintained below 10% and 8%, respectively. Furthermore, the respective quantifiable ranges for these cytokines were established as 15.63–1,000 pg/mL for IL-1β, 7.82–500 pg/mL for IL-6, 15.63–1,000 pg/mL for TNF-α, and 31.25–2,000 pg/mL for MCP-1, ensuring a high degree of precision and sensitivity in the measurements obtained.

### 2.4 Histological analysis

The fresh liver tissue was immersed in 4% formaldehyde solution for 24 h for fixation. Following fixation, the tissue underwent dehydration through a series of graded ethanol concentrations, was subsequently embedded in paraffin, and sectioned into 4-μm slices. These sections were subsequently stained with hematoxylin and eosin (H&E) to facilitate histological assessment of hepatic injury. The degree of hepatic injury was meticulously evaluated utilizing the Suzuki scoring system, which provides a standardized metric for assessing histopathological alterations in liver tissue. This system quantitatively assesses parameters such as congestion, ballooning degeneration, and necrosis, thereby offering a comprehensive evaluation of liver damage severity. The following are its specific contents: Hepatocellular congestion (0 points: No congestion; 1 point: The range of congestion is less than 10%; 2 points: congestion range of 10%–30%; 3 points: congestion range of 31%–60%; 4 points: The range of congestion is greater than 60%.), The ballooning degeneration of liver cells (0 points: No ballooning degeneration; 1 point: The range of variation of the ballooning degeneration is less than 10%; 2 points: the ballooning degeneration variation range of 10%–30%; 3 points: the ballooning degeneration variation range of 31%–60%; 4 points: The range of variation of the ballooning degeneration is greater than 60%) and Hepatocyte necrosis (0 points: No necrosis; 1 point: Single cell necrosis; 2 points: Necrosis range less than 30%; 3 points: Necrosis range of 31%–60%; 4 points: Necrosis range greater than 60%). Each parameter is scored separately, and then the scores of the three parameters are added together to obtain the total score. The total score ranges from 0 to 12 points, with higher scores indicating more severe liver damage.

### 2.5 Real-time quantitative PCR

RNA was extracted from liver tissue using the Trizol reagent (Invitrogen, NY, United States). The concentration of RNA was determined using a NanoDrop ND-1000 spectrophotometer (Thermo Fisher Scientific, MA, United States). The RNA was then reverse-transcribed into cDNA using a reverse transcription kit (Vazyme, Nanjing, China). The expression of the target genes was assessed using SYBR Green in a quantitative real-time PCR (qPCR) assay. The relative fold changes between the experimental and calibrator samples were calculated using the 2^−ΔΔCt^ method (Livak method). The primer sequences are presented in Table 1.

**TABLE 1:** Primer sequence for quantitative real-time PCR.

Gene		Sequences	NCBI gene ID	Amplicon Size
*18S*	Forward	AGG​TCT​GTG​ATG​CCC​TTA​GA	19791	109
	Reverse	GAA​TGG​GGT​TCA​ACG​GGT​TA		
*Bcl2*	Forward	GCG​GAG​TTC​ACA​GCT​CTA​TAC	12043	136
	Reverse	AAA​AGG​CCC​CTA​CAG​TTA​CCA		
*Bax*	Forward	TGA​AGA​CAG​GGG​CCT​TTT​TG	12028	140
	Reverse	AAT​TCG​CCG​GAG​ACA​CTC​G		
*Grp78*	Forward	GCA​TCA​CGC​CGT​CGT​ATG​T	14828	134
	Reverse	ATT​CCA​AGT​GCG​TCC​GAT​GAG		
*Atf4*	Forward	AAC​CTC​ATG​GGT​TCT​CCA​GCG​A	11911	125
	Reverse	CTC​CAA​CAT​CCA​ATC​TGT​CCC​G		
*Chop*	Forward	CCC​TCG​CTC​TCC​AGA​TTC​C	13198	131
	Reverse	TCT​CCT​TCA​TGC​GTT​GCT​T		
*Gpx4*	Forward	GCC​TGG​ATA​AGT​ACA​GGG​GTT	625249	99
	Reverse	CAT​GCA​GAT​CGA​CTA​GCT​GAG		

### 2.6 Western blotting

Western blotting experiments were conducted as previously described (Ding et al., 2022). In brief, liver tissue was homogenized using RAPI lysis buffer (Boster, Wuhan, China). Proteins were resolved by SDS-PAGE and subsequently transferred onto PVDF membranes. The membranes were then blocked with a 5% solution of skim milk to prevent non-specific binding. Following this, the membranes were incubated with their respective primary antibodies at 4°C for an extended period, typically overnight. After three rinses with Tris-buffered saline with Tween 20 (TBST) to remove unbound antibodies, the membranes were further incubated with horseradish peroxidase (HRP)-tagged secondary antibodies at ambient temperature for 1 h. The detection of protein expression was achieved by chemiluminescence using an enhanced chemiluminescence (ECL) detection kit (Vazyme, Nanjing, China). The optical densities of the bands were subsequently quantified employing ImageJ software, providing a numerical representation of the immunoreactive bands. The following primary antibodies were used: BCL-2 (1:1,000, #ET1702-53), BAX (1:1,000, #ET1603-34), ATF4 (1:1,000, #ET1612-37)—all from HUABIO, China; GRP78 (1:1,000, #AF5366), p-PERK (1:1,000, #DF7576), p-eIF2α (1:1,000, #AF3087), CHOP (1:1000, #AF6277) —all from Affinity Biosciences, China; GPX4 (1:1,000, #BM5231), eIF2α (1:1,000, #M04387-5)—all from Boster, China; PERK (1:1,000, #24390-1-AP, Protenintech, China); β-actin (1:20,000, #AC026, ABclonal, China). The following secondary antibodies were used: HRP Conjugated Affini Pure Goat Anti-mouse IgG (H + L) (1:5,000, #BA1050), HRP Conjugated Affini Pure Goat Anti-rabbit IgG (H + L) (1:5,000, #BA1054)—all from Boster, China.

### 2.7 Statistical analysis

All data were expressed as the mean ± the standard deviation (SD). Upon conducting the Shapiro-Wilk test and the Kolmogorov-Smirnov test, the data distribution was found to conform to a normal distribution. Statistical evaluations were performed using one-way analysis of variance (ANOVA), complemented by Tukey’s *post hoc* test for pairwise comparisons. The Graph Pad Prism software (version 8.0.1) was employed utilized to visualize the results. *P* < 0.05 was considered statistical significance in all experimental analyses.

## 3 Results

### 3.1 TA ameliorates APAP-induced liver injury in mice

**FIGURE 1:**
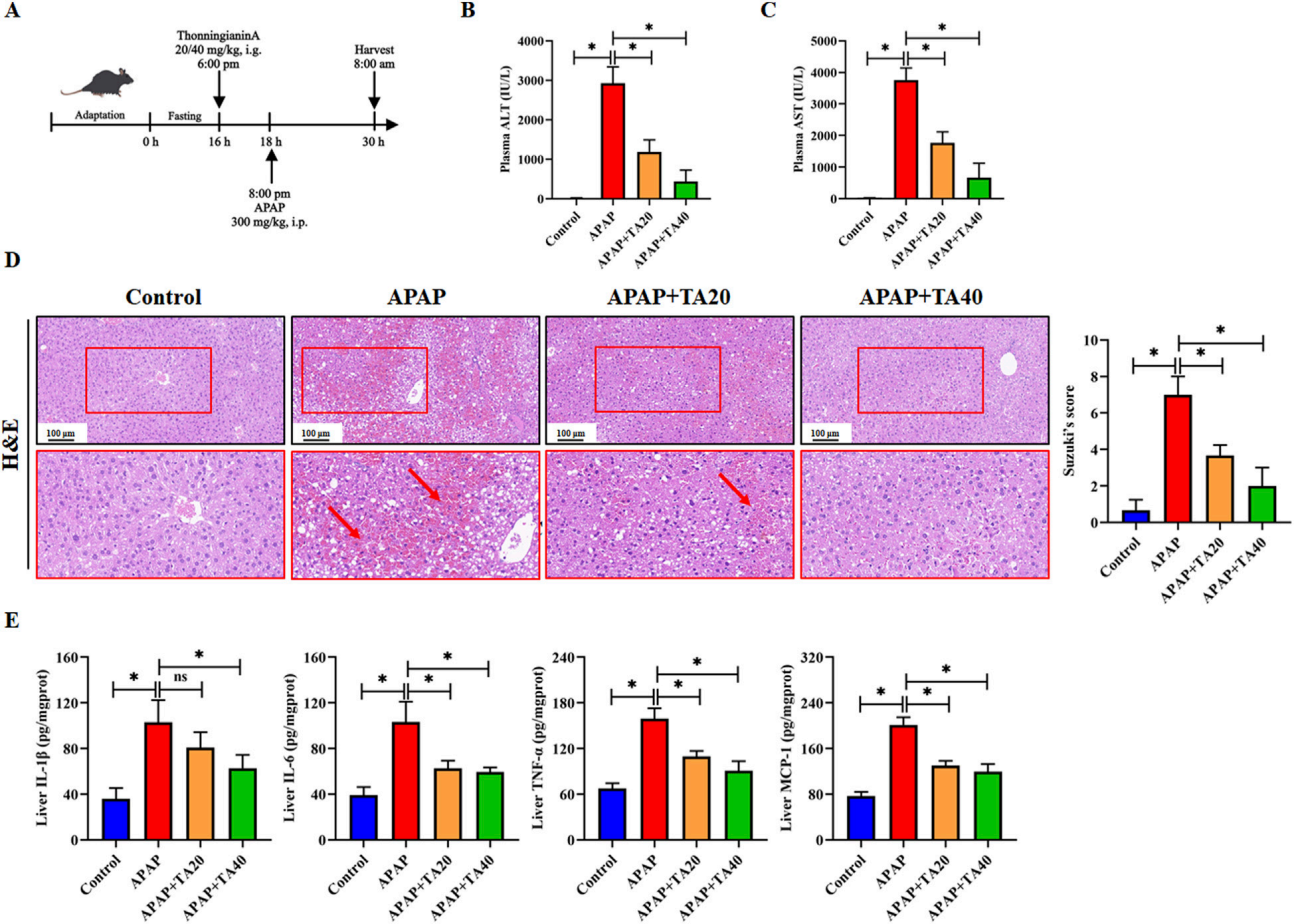
TA ameliorates APAP-induced liver injury in mice. **(A)** Schematic Diagram of the Animal Experiment. Fasted for 16 h, mice were administered TA [20 or 40 mg/kg, i.g.] 2 h prior to saline or APAP (300 mg/kg, i.p.) for 12 h. **(B)** Plasma levels of ALT. **(C)** Plasma levels of AST. **(D)** Liver tissues were subjected to H&E staining for histological examination. **(E)** Concentrations of hepatic IL-1β, IL-6, TNF-α, and MCP-1 were quantified using commercially available ELISA kits. All data were presented as mean ± SD, n = 5–8 per group. *P < 0.05 vs. corresponding control.

To investigate the protective effect of TA against APAP-induced liver injury, C57BL/6 mice were fasted. Then they administered an intraperitoneal injection of APAP (300 mg/kg), with oral doses of TA (20 and 40 mg/kg) given 2 h prior (Figure 1A). Compared to the control group, plasma ALT and AST levels were significantly elevated 12 h after APAP injection. However, TA treatment markedly reduced serum ALT and AST levels (Figures 1B,C). Histopathological analysis using H&E staining revealed pronounced ballooning degeneration and cytoplasmic vacuolization in the APAP group, which were substantially alleviated by TA treatment (Figure 1D). Moreover, enzyme-linked immunosorbent assay (ELISA) results demonstrated that TA significantly decreased hepatic levels of IL-1β, IL-6, TNF-α, and MCP-1 (Figure 1E). In summary, these findings highlight the hepatoprotective effects of TA in a dose-dependent manner in ALI mouse models.

### 3.2 TA inhibits APAP-induced hepatocellular apoptosis

**FIGURE 2:**
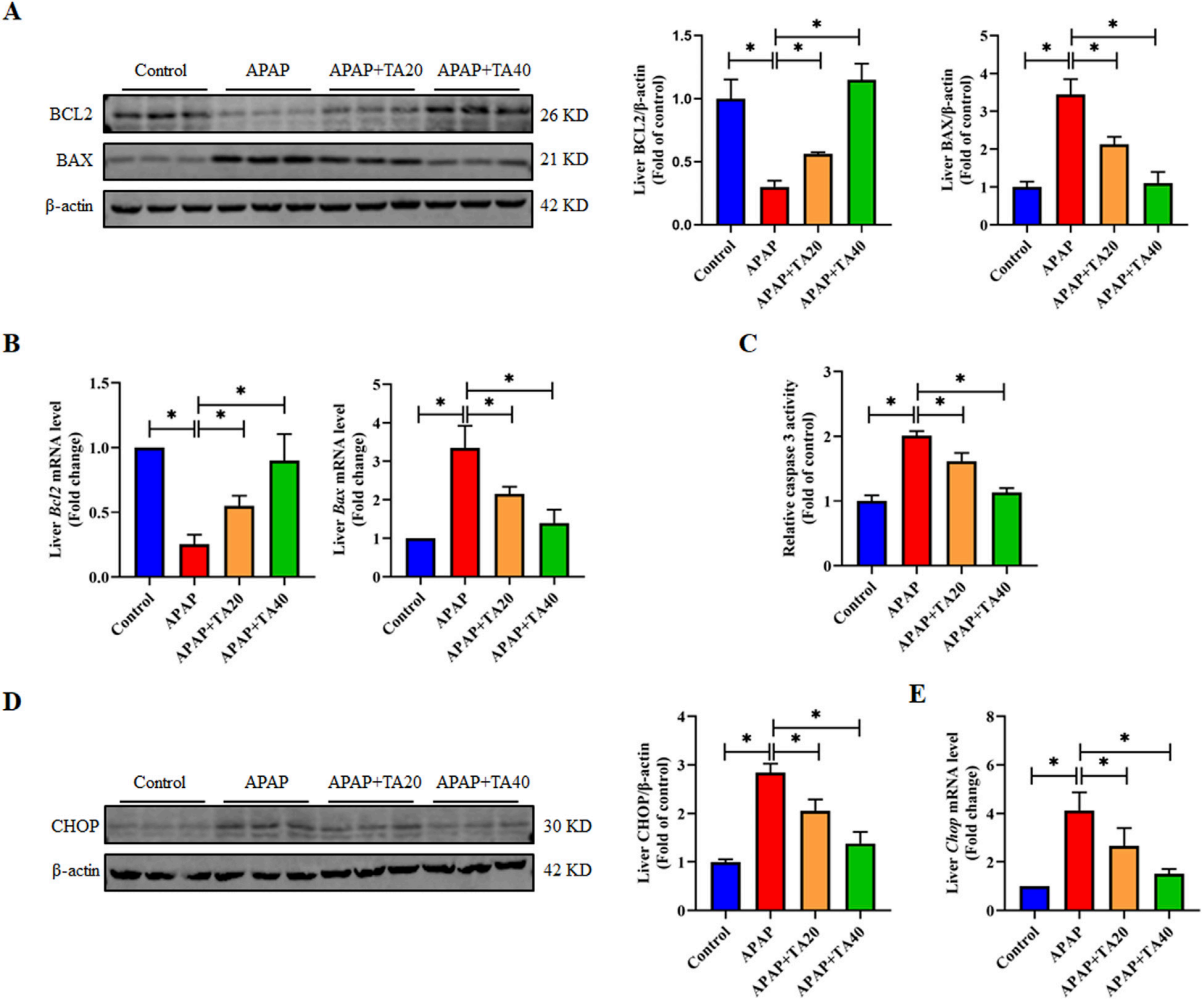
TA inhibits APAP-induced hepatocellular apoptosis. **(A)** Western blotting assessed hepatic BCL2 and BAX expression, with β-actin as a loading control. Band intensities were quantified using ImageJ. **(B)** q-PCR was conducted to evaluate the transcript levels of BCL2 and BAX-related genes. **(C)** Hepatic caspase 3 activity was quantified using a commercially available kit (fold of control). **(D)** Western blotting assessed hepatic CHOP expression, with β-actin as a loading control. Band intensities were quantified using ImageJ. **(E)** q-PCR was conducted to evaluate the transcript levels of the *Chop* gene. All data were presented as mean ± SD, n = 3 for Western blotting, n = 5–8 for others. *P < 0.05 vs. corresponding control.

Hepatocyte apoptosis is a crucial feature of ALI (Zhou et al., 2021). To further explore the impact of TA on hepatocyte apoptosis, we analyzed the expression of several apoptosis-related proteins and genes using Western blotting and RT-PCR. The results showed that APAP significantly downregulated the protein and mRNA levels of the anti-apoptotic gene BCL2 and upregulated those of the pro-apoptotic gene BAX. These effects were reversed by TA treatment (Figures 2A,B). Additionally, Caspase-3, an enzyme central to the apoptotic process and a commonly used biomarker of apoptosis displayed significantly increased activity in the APAP group compared to controls. TA treatment notably reduced Caspase-3 activity, compared to the model group, treatment with TA (40 mg/kg) resulted in a reduction of Caspase-3 activity by approximately 50% (Figure 2C). Furthermore, the C/EBP homologous protein (CHOP), a transcription factor that can promote Caspase-3 activation and drive apoptosis when upregulated, exhibited significantly elevated protein and mRNA levels in the APAP group. This effect was reversed by TA treatment (Figures 2D,E). In conclusion, these findings suggest that TA mitigates APAP-induced hepatocyte apoptosis.

### 3.3 TA mitigates APAP-induced hepatotoxicity through the alleviation of ER stress

**FIGURE 3:**
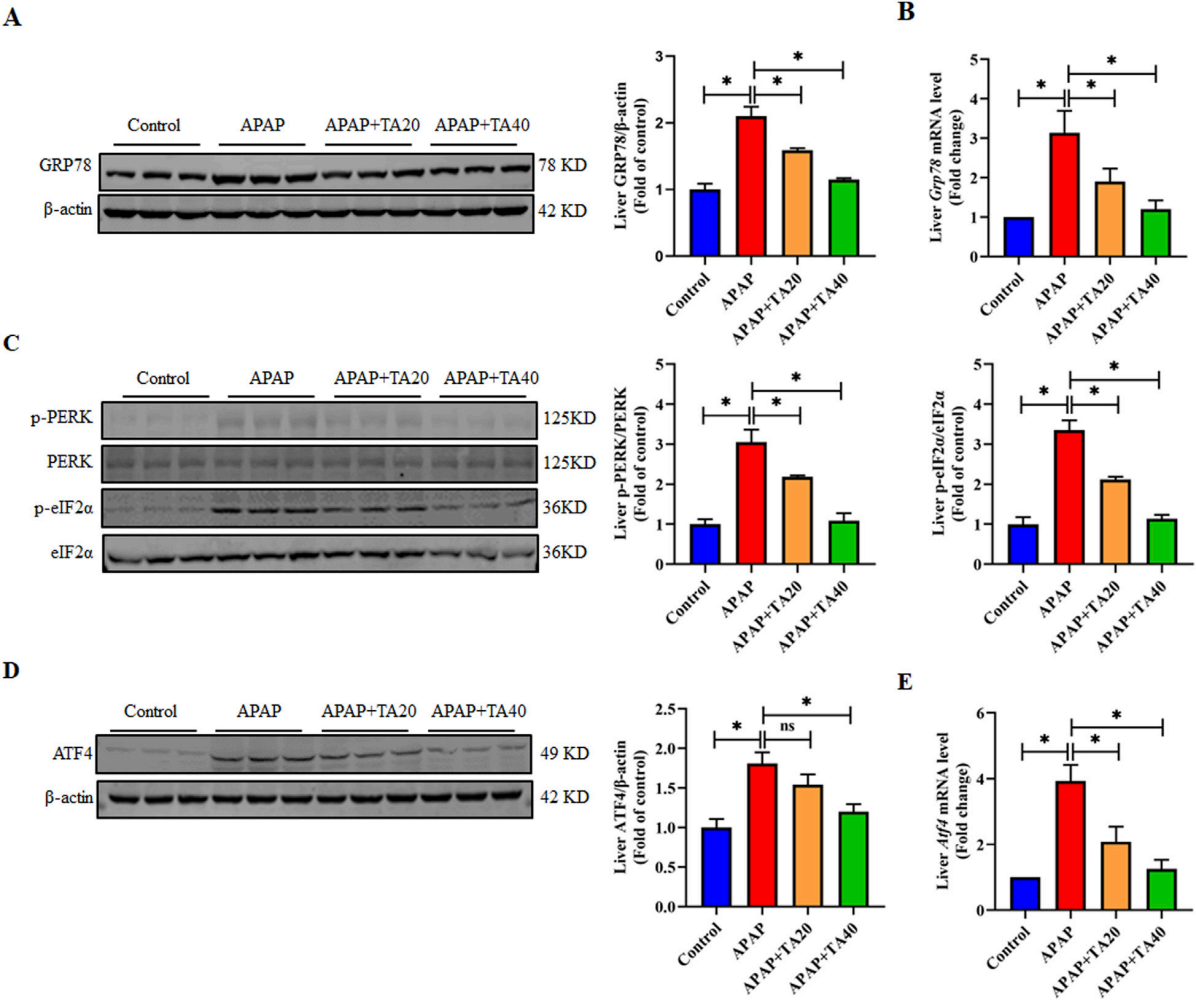
TA mitigates APAP-induced hepatotoxicity through the alleviation of ER stress. **(A)** Western blotting assessed hepatic GRP78 expression, with β-actin as a loading control. Band intensities were quantified using ImageJ. **(B)** q-PCR was conducted to evaluate the transcript levels of the *Grp78* gene. **(C)** Western blotting assessed hepatic phosphorylated-PERK, phosphorylated-eIF2α, PERK, and eIF2α protein expressions. Band intensities were quantified using ImageJ. **(D)** Western blotting assessed hepatic ATF4 expression, with β-actin as a loading control. Band intensities were quantified using ImageJ. **(E)** q-PCR was conducted to evaluate the transcript levels of the *Atf4* gene. All data were presented as mean ± SD, n = 3 for Western blotting, n = 6 for others. *P < 0.05 vs. corresponding control.

CHOP plays a pivotal role in ER stress-mediated apoptosis (Qin et al., 2022). ER stress occurs in response to the accumulation of misfolded or unfolded proteins in the ER, a key mechanism in APAP-induced liver injury. Therefore, we investigated whether TA treatment modulates the ER stress signaling pathway. Western blotting and RT-PCR results demonstrated that TA downregulated the protein and mRNA expression levels of the ER stress marker GRP78, which were elevated by APAP treatment (Figures 3A,B). Additionally, TA intervention reduced the expression levels of p-PERK, p-eIF2α, and ATF4 (Figures 3C–E), indicating that TA affects the PERK-eIF2α-ATF4 branch of the ER stress response. Collectively, these findings suggest that TA alleviates APAP-induced ER stress in the liver. In conclusion, this study demonstrates that TA modulates key proteins in the ER stress response, contributing to mitigating APAP-induced hepatotoxicity.

### 3.4 TA mediates the enhancement of hepatic antioxidant function by GPX4

**FIGURE 4:**
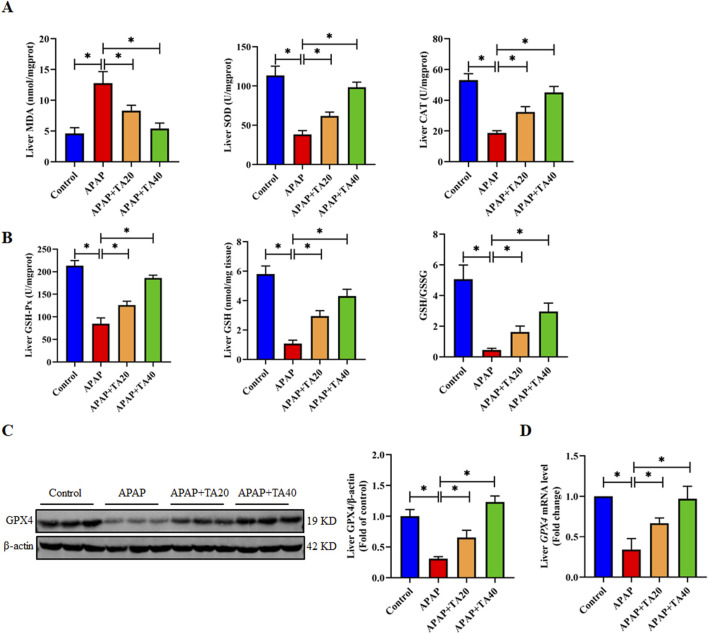
TA mediates the enhancement of hepatic antioxidant function by GPX4. **(A)** Hepatic concentrations of MDA, alongside the enzymatic activities of SOD and CAT. **(B)** Hepatic concentrations of GSH-Px, GSH, and the GSH redox ratio (GSH/GSSG) were determined. **(C)** Western blotting assessed hepatic GPX4 expression, with β-actin as a loading control. Band intensities were quantified using ImageJ. **(D)** q-PCR was conducted to evaluate the transcript levels of the *Gpx4* gene. All data were presented as mean ± SD, n = 3 for Western blotting, n = 6–8 for others. *P < 0.05 vs. corresponding control.

Oxidative stress is one of the important inducers of endoplasmic reticulum stress (Zhang et al., 2022). Research indicates that an overdose of APAP leads to the accumulation of its toxic metabolite N-acetyl-p-benzoquinone imine (NAPQI), which triggers oxidative stress in liver tissue (Chowdhury et al., 2020). To investigate the protective effects of TA on APAP-induced oxidative liver injury, we measured the levels of MDA, SOD, CAT, GSH-Px, and GSH in liver tissue. The results demonstrated a significant increase in MDA levels, accompanied by a marked decrease in GSH levels, as well as the activities of SOD, CAT, GSH-Px, and the GSH redox ratio (GSH/GSSG) in the APAP-treated group. TA treatment significantly reversed these oxidative stress markers (Figures 4A,B). Given the role of GPX4 in maintaining redox homeostasis and its connection to ALI, we further assessed whether TA mitigates APAP-induced hepatotoxicity through modulation of the GPX4 pathway. Western blotting and RT-PCR analyses revealed a substantial decrease in GPX4 protein and mRNA expression in the APAP group, significantly restored by TA treatment (Figures 4C,D). This suggests that TA alleviates APAP-induced liver injury by enhancing GPX4-mediated antioxidant defense mechanisms.

### 3.5 Liver-specific GPX4 knockdown abrogates the hepatoprotective effects of TA against APAP-induced hepatotoxicity

**FIGURE 5:**
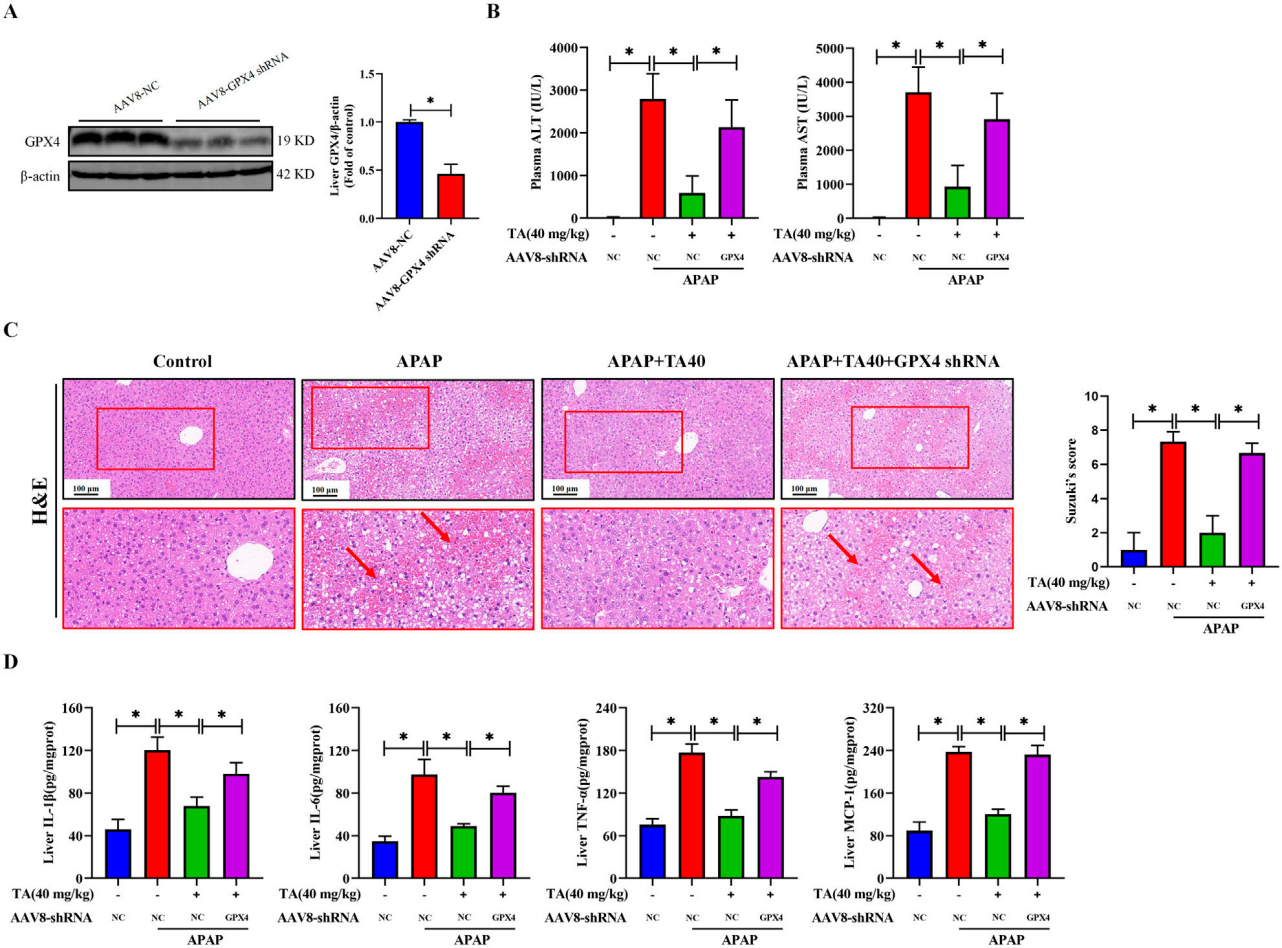
Liver-specific GPX4 knockdown abrogates the hepatoprotective effects of TA against APAP-induced hepatotoxicity. **(A)** Western blotting assessed the detected hepatic GPX4 knockdown efficiency in protein levels. Hepatocyte-specific GPX4 knockdown mice were created by AAV8-mediated delivery of a TBG promoter-driven shRNA targeting GPX4. Null-vector-injected mice served as control. **(B)** Plasma levels of ALT and AST. **(C)** Liver tissues were subjected to H&E staining for histological examination. **(D)** Concentrations of hepatic IL-1β, IL-6, TNF-α, and MCP-1 were quantified using commercially available ELISA kits. All data were presented as mean ± SD, n = 3 for Western blotting, n = 5 for others. *P < 0.05 vs. corresponding control.

To ascertain the role of GPX4 in the ameliorative effects of TA against ALI in mice, we utilized liver-specific GPX4 knockdown mice. This was achieved by injecting recombinant adeno-associated virus serotype 8 (AAV8) vectors containing a hepatocyte-specific promoter and GPX4 shRNA into the lateral tail vein of mice. Western blot analysis revealed a 53.6% reduction in hepatic GPX4 protein levels (Figure 5A), confirming the successful establishment of the hepatocyte-specific GPX4 knockdown (GPX4 KD) model. Compared to the APAP + TA group, serum ALT and AST levels were significantly elevated in the APAP + TA + GPX4 KD group (Figure 5B). Histological analysis via H&E staining further showed that GPX4 knockdown partially abrogated the protective effects of TA (Figure 5C). Additionally, ELISA results revealed that GPX4 knockdown significantly reversed TA’s anti-inflammatory effects, as indicated by increased hepatic levels of IL-1β, IL-6, TNF-α, and MCP-1 in the APAP + TA + GPX4 KD group compared to the APAP + TA group (Figure 5D). Collectively, these findings demonstrate that GPX4 plays a critical role in the hepatoprotective effects of TA against APAP-induced liver injury. In conclusion, TA modulates the expression of key proteins involved in the ER stress response, mitigating APAP-induced hepatotoxicity.

### 3.6 Liver-specific GPX4 knockdown abrogates the protective effect of TA against APAP-induced hepatocyte apoptosis

**FIGURE 6:**
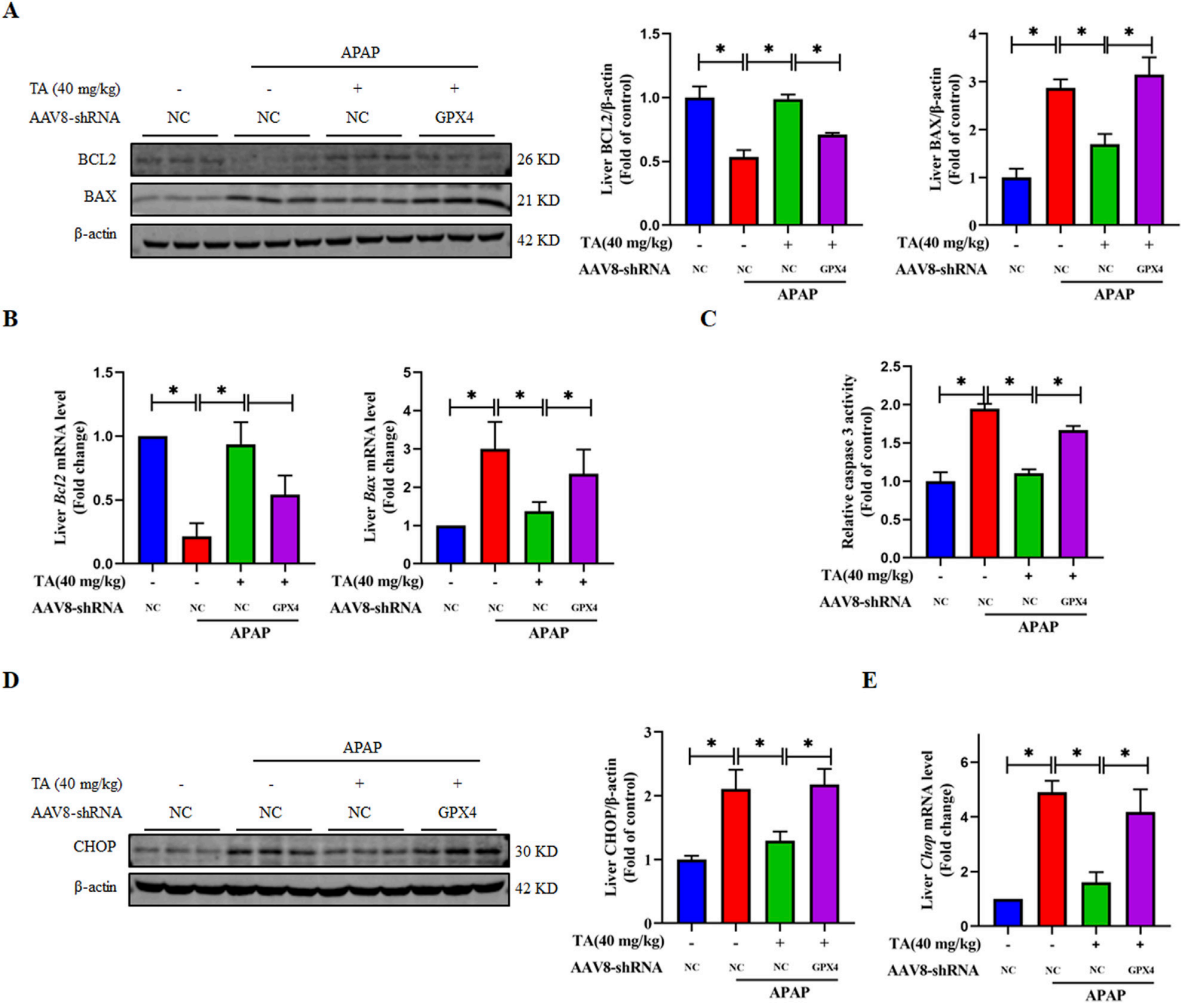
Liver-specific GPX4 knockdown abrogates the protective effect of TA against APAP-induced hepatocyte apoptosis. **(A)** Western blotting assessed hepatic BCL2 and BAX expression, with β-actin as a loading control. Band intensities were quantified using ImageJ. **(B)** q-PCR was conducted to evaluate the transcript levels of BCL2 and BAX-related genes. **(C)** Hepatic caspase 3 activity was quantified using a commercially available kit (fold of control). **(D)** Western blotting assessed hepatic CHOP expression, with β-actin as a loading control. Band intensities were quantified using ImageJ. **(E)** q-PCR was conducted to evaluate the transcript levels of the *Chop* gene. All data were presented as mean ± SD, n = 3 for Western blotting, n = 5 for others. *P < 0.05 vs. corresponding control.

To further investigate the role of liver-specific GPX4 knockdown in the protective effects of TA against APAP-induced apoptosis, we analyzed the expression of key pro-apoptotic and anti-apoptotic proteins in the APAP + TA and APAP + TA + GPX4 KD groups. The pro-apoptotic protein BAX was significantly upregulated, while the anti-apoptotic protein BCL2 was markedly downregulated at both the protein and mRNA levels in the GPX4 KD group (Figures 6A,B). In addition, the activity of the apoptotic protein Caspase 3 was significantly elevated in GPX4 KD mice. Relative to the TA-treated group, the GPX4 shRNA group exhibited an approximately 1.5-fold enhancement in activity (Figure 6C). Furthermore, the reduction in CHOP protein and mRNA levels induced by TA was reversed in the GPX4 KD group (Figures 6D, E). These findings collectively suggest that GPX4 is critical for the protective effects of TA against ALI, as its knockdown abrogates the modulation of apoptosis-related proteins and Caspase 3 activity by TA.

### 3.7 Liver-specific GPX4 knockdown inhibits TA-regulated ER stress in APAP-induced hepatotoxicity

**FIGURE 7:**
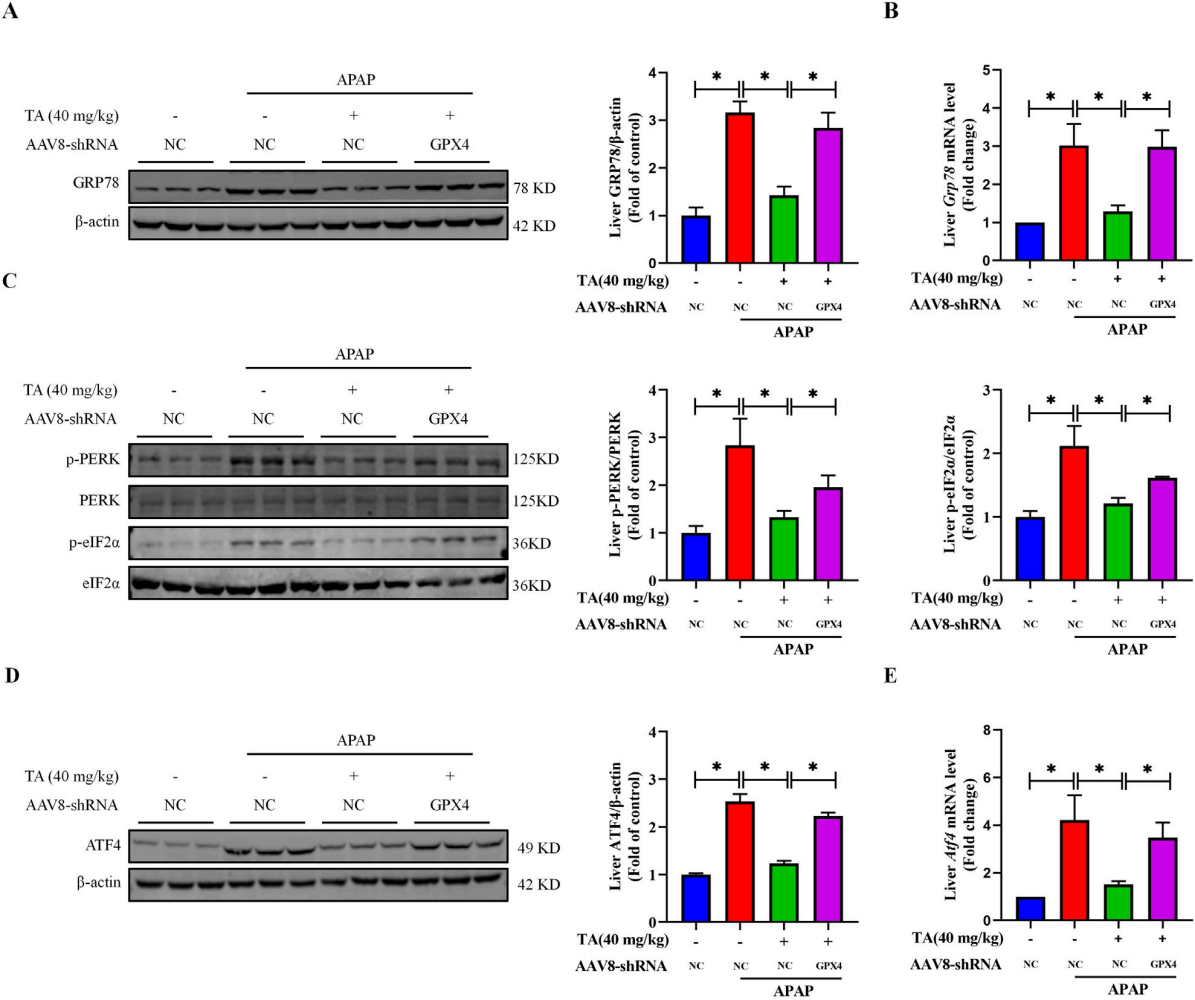
Liver-specific GPX4 knockdown inhibits TA-regulated ER stress in APAP-induced hepatotoxicity. **(A)** Western blotting assessed hepatic GRP78 expression, with β-actin as a loading control. Band intensities were quantified using ImageJ. **(B)** q-PCR was conducted to evaluate the transcript levels of the *Grp78* gene. **(C)** Western blotting assessed hepatic phosphorylated-PERK, phosphorylated-eIF2α, PERK, and eIF2α protein expressions. Band intensities were quantified using ImageJ. **(D)** Western blotting assessed hepatic ATF4 expression, with β-actin as a loading control. Band intensities were quantified using ImageJ. **(E)** q-PCR was conducted to evaluate the transcript levels of the *Atf4* gene. All data were presented as mean ± SD, n = 3 for Western blotting, n = 5 for others. *P < 0.05 vs. corresponding control.

To further explore the role of GPX4 in TA’s protective effects against ER stress, we analyzed the expression of ER stress-related proteins and genes using Western blotting and RT-PCR. The results demonstrated that, compared to the APAP + TA group, the expression levels of GRP78, p-PERK, p-eIF2α, and ATF4 were significantly elevated in the APAP + TA + GPX4 KD group (Figure 7). This confirms the involvement of the PERK-eIF2α-ATF4 signaling pathway in TA’s hepatoprotective effects against ALI. The upregulation of these ER stress markers in GPX4 KD mice after TA treatment suggests that GPX4 is crucial for mediating TA’s effects.

## 4 Discussion

**FIGURE 8:**
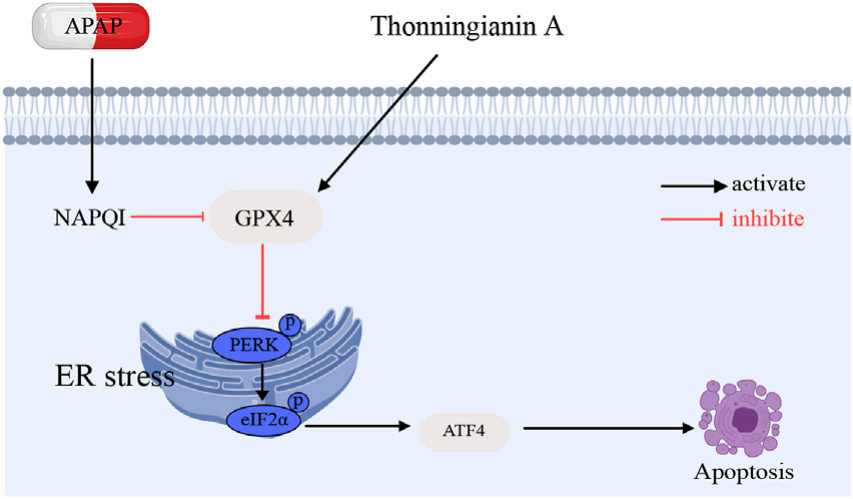
Schematic illustration of the protective effects and mechanisms of TA APAP-induced liver injury.

In this study, we demonstrated the significant protective effects of TA against APAP-induced liver injury by mitigating oxidative and ER stress, with GPX4 playing a crucial regulatory role. The findings confirm that APAP overdose triggers severe oxidative stress by depleting GSH and generating excessive ROS, which subsequently disrupts ER homeostasis, activating UPR pathways such as the PERK-ATF4 axis. This exacerbates ER stress and contributes to hepatocyte apoptosis in ALI. Notably, TA treatment alleviates these detrimental effects by modulating oxidative stress and regulating the UPR, thereby improving APAP-induced liver injury (Figure 8).

Apoptosis, a form of programmed cell death, plays a critical role in ALI. Excessive intake of APAP impairs the formation of apoptotic bodies (Wan et al., 2020). This process activates Caspase 3 while reducing the expression of the anti-apoptotic protein BCL-2 and increasing the expression of the pro-apoptotic protein BAX (Wang et al., 2022). Our study demonstrates that treatment with TA significantly decreased Caspase 3 activity and increased BCL-2 expression while decreasing BAX expression. These results suggest that TA exerts anti-apoptotic effects in ALI by modulating Caspase 3 activity and the expression of BCL-2 family proteins. CHOP is a crucial regulator of ER stress-induced apoptosis. It promotes apoptosis when overexpressed and protects against apoptosis when knocked down (Wang et al., 2021; Li J. et al., 2024). Our results demonstrate that APAP significantly increased hepatic CHOP expression, whereas TA treatment markedly reversed this effect. These findings suggest that the reduction in hepatocyte apoptosis observed with TA treatment is closely associated with the modulation of CHOP. Furthermore, TA appears to downregulate CHOP through the modulation of the PERK-eIF2α-ATF4 signaling pathway. The activation of this pathway under ER stress conditions leads to CHOP upregulation, which promotes apoptosis. However, TA treatment effectively attenuates this pathway, reducing the levels of CHOP and thereby preventing the apoptotic cascade in hepatocytes.

ER stress and apoptosis are closely interlinked processes, particularly in the context of APAP-induced liver injury (Shu Y. et al., 2020; Fernandez-Checa et al., 2021). The persistent activation of ER stress can result in apoptosis, primarily mediated by CHOP (Zhang et al., 2022). This key transcription factor is upregulated during prolonged ER stress and promotes cell death through the induction of pro-apoptotic pathways (Rozpedek et al., 2016). Previous studies have demonstrated that the PERK-eIF2α-ATF4 axis, a key component of the unfolded protein response (UPR) activated during endoplasmic reticulum (ER) stress, is upregulated in ALI (Chen et al., 2019; Li et al., 2020; Xu et al., 2023). This pathway is a key component of the UPR. PERK phosphorylates eIF2α, resulting in decreased protein synthesis and increased translation of ATF4. ATF4, a critical transcription factor, regulates CHOP during ER stress and is involved in various cellular processes, including amino acid metabolism and cell survival (Yang et al., 2021; van der Mijn et al., 2022). Our study demonstrated that APAP-induced ER stress led to significant upregulation of CHOP alongside other ER stress markers such as GRP78, p-PERK, p-eIF2α, and ATF4. These changes are part of the PERK-eIF2α-ATF4 signaling pathway, crucial in the ER stress response and subsequent apoptotic signaling. Importantly, TA treatment significantly suppressed the expression of these markers, indicating its potential to alleviate ER stress in the context of APAP-induced liver injury. By modulating the activation of the PERK-eIF2α-ATF4 axis, TA reduced the p-PERK and the upregulation of ATF4, which are typically elevated during ER stress. Additionally, TA reduced the expression of GRP78, an ER chaperone protein that is upregulated in response to stress. These findings suggest that TA exerts its protective effects by modulating the activation of the UPR and the downstream signaling cascade, helping to restore cellular homeostasis and prevent apoptotic pathways triggered by sustained ER stress.

Moreover, our results show that the protective effects of TA are closely linked to the antioxidant enzyme GPX4, which plays a pivotal role in mitigating lipid peroxidation and oxidative stress. MDA, a key end product of lipid peroxidation, serves as a widely recognized biomarker for oxidative stress (Jelic et al., 2021). GPX4 is crucial for reducing lipid peroxides by catalyzing their conversion into alcohols, thus preventing the propagation of oxidative damage. Consequently, GPX4 activity exhibits an inverse relationship with MDA levels: when GPX4 is functioning optimally, MDA production is reduced, alleviating cellular oxidative stress (Forcina and Dixon, 2019). TA significantly upregulated GPX4 expression in the liver tissues of APAP-treated mice, aligning with a reduction in MDA levels, which suggests a decrease in lipid peroxidation and overall oxidative stress. Furthermore, GPX4 works synergistically with other antioxidant enzymes such as SOD and CAT. SOD catalyzes the conversion of superoxide anions to hydrogen peroxide, and GPX4 helps to further reduce hydrogen peroxide into water, thereby supporting redox homeostasis (Buettner, 2011; Conrad and Sato, 2012). CAT, predominantly located in peroxisomes, decomposes hydrogen peroxide into water and oxygen, working in concert with GPX4 to mitigate oxidative damage. Together with GSH-Px, a component of the glutathione antioxidant system, GPX4 utilizes reduced GSH to reduce lipid peroxides, contributing to the overall antioxidant defense (Pei et al., 2023). In summary, the regulation of lipid peroxidation by GPX4 is essential for managing oxidative stress, and its activity directly influences the levels of key oxidative stress markers. The upregulation of GPX4 by TA not only reduces lipid peroxides but also enhances the activity of key antioxidant enzymes like SOD and CAT, underscoring GPX4’s critical role in TA’s protective effects against APAP-induced liver injury.

Most notably, our finding that the knockdown of GPX4 disrupts TA regulation of ER stress supports a protective role for GPX4 in the cellular stress response. Reduced GPX4 expression amplified markers of ER stress, including elevated levels of GRP78, p-PERK, and ATF4. This suggests that GPX4 deficiency disrupts cellular redox homeostasis, leading to an exacerbation of the ER stress response. This finding is consistent with the established role of GPX4 in preventing lipid peroxidation within ER membranes. The elevated ER stress observed in the GPX4 knockout group emphasizes the interconnection between oxidative stress and ER stress (Lee, 2020; Zhao et al., 2021; Huang et al., 2024), as GPX4 deficiency may lead to the accumulation of ROS, which further exacerbates ER dysfunction and the accumulation of misfolded proteins (Iuchi et al., 2021; Jehan et al., 2022; Zhang et al., 2022). Our findings suggest that the efficacy of TA in ameliorating ER stress depends, at least in part, on the expression of GPX4. When GPX4 was knocked down, the ability of TA to reduce ER stress markers (e.g., GRP78, p-PERK, and ATF4) was markedly attenuated, suggesting that GPX4 is a key mediator of the protective effects of TA. Additionally, the knockdown of GPX4 led to a significant increase in ALT and AST levels, suggesting a loss of liver function and further supporting the critical role of GPX4 in protecting liver integrity. This was further corroborated by histological examination, which revealed more severe liver damage, in the GPX4 knockdown group compared to the TA-treated control group. These findings highlight the importance of GPX4 in preserving liver function and in the protective effects of TA against APAP-induced liver injury.

Recent studies have demonstrated that TA enhances GPX4 activity and expression through multiple mechanisms, including activation of the AMPK/Nrf2 signaling pathway (Yong et al., 2024). This pathway upregulates antioxidant enzymes, bolstering the cellular defense against oxidative stress and mitigating oxidative damage. Additionally, GPX4 plays a central role in inhibiting ferroptosis, a form of cell death characterized by excessive lipid peroxidation. TA’s ability to upregulate GPX4 expression indirectly suppresses ferroptosis, thereby protecting cells from ferroptotic damage (Yong et al., 2024). Moreover, TA’s activation of the Keap1-Nrf2 pathway further enhances GPX4’s antioxidant function and reinforces cellular redox homeostasis (Sun et al., 2022). Beyond these direct effects, TA may also regulate other stress-related and apoptotic pathways, suggesting that GPX4 interacts with multiple molecular pathways to maintain cellular integrity. Together, these mechanisms form the molecular basis for TA’s protective effects, with GPX4 acting as a critical regulator of oxidative stress, ferroptosis, and cellular stress responses.

These observations provide novel insights into the interplay between oxidative stress and ER stress, highlighting GPX4 as a pivotal regulator of both processes. The data suggest that GPX4 contributes to the direct suppression of lipid peroxidation and oxidative stress and plays an essential role in modulating the PERK-ATF4 signaling pathway in response to ER stress. Furthermore, the loss of GPX4 impairs the ability of pharmacological interventions like TA to effectively mitigate ER stress, underlining the importance of GPX4 in maintaining cellular homeostasis under conditions of stress. Despite the promising findings of this study, several limitations should be acknowledged to provide a balanced perspective. First, while we demonstrated that GPX4 plays a pivotal role in TA’s protective effects, the precise molecular mechanisms through which GPX4 modulates ER stress and oxidative stress remain incompletely understood. For instance, the interaction of GPX4 with specific signaling molecules or pathways, such as PERK-ATF4 and CHOP, warrants further investigation to elucidate its broader regulatory roles. Second, the potential for TA to act on other antioxidant or anti-inflammatory pathways beyond GPX4 has not been fully explored in this study. Future research should aim to comprehensively evaluate potential off-target effects of TA and its interaction with other cellular defense mechanisms.

## 5 Conclusion

In summary, this research expands our understanding of the mechanisms underlying APAP-induced liver injury and highlights the importance of GPX4 in modulating stress response pathways. The study also provides a foundation for exploring GPX4 as a therapeutic target in liver diseases characterized by oxidative and ER stress. Future studies should investigate the potential of GPX4 activators or other pharmacological agents to further enhance the protective effects of treatments like TA, providing new avenues for the prevention and treatment of drug-induced liver injury.

## Data Availability

The raw data supporting the conclusions of this article will be made available by the authors, without undue reservation.

## References

[B1] Ajoolabady A. Kaplowitz N. Lebeaupin C. Kroemer G. Kaufman R. J. Malhi H. (2023). Endoplasmic reticulum stress in liver diseases. Hepatology 77 (2), 619–639. 10.1002/hep.32562 35524448 PMC9637239

[B2] Buettner G. R. (2011). Superoxide dismutase in redox biology: the roles of superoxide and hydrogen peroxide. Anticancer Agents Med. Chem. 11 (4), 341–346. 10.2174/187152011795677544 21453242 PMC3131414

[B3] Cai X. Cai H. Wang J. Yang Q. Guan J. Deng J. (2022). Molecular pathogenesis of acetaminophen-induced liver injury and its treatment options. J. Zhejiang Univ. Sci. B 23 (4), 265–285. 10.1631/jzus.B2100977 35403383 PMC9002247

[B4] Chen Y. Guan S. Guan Y. Tang S. Zhou Y. Wang X. (2022). Novel clinical biomarkers for drug-induced liver injury. Drug Metab. Dispos. 50 (5), 671–684. 10.1124/dmd.121.000732 34903588

[B5] Chen Y. Park H. J. Park J. Song H. C. Ryter S. W. Surh Y. J. (2019). Carbon monoxide ameliorates acetaminophen-induced liver injury by increasing hepatic HO-1 and Parkin expression. FASEB J. 33 (12), 13905–13919. 10.1096/fj.201901258RR 31645120

[B6] Cheng X. M. Hu Y. Y. Yang T. Wu N. Wang X. N. (2022). Reactive oxygen species and oxidative stress in vascular-related diseases. Oxid. Med. Cell. Longev. 2022, 7906091. 10.1155/2022/7906091 35419169 PMC9001081

[B7] Chilvery S. Yelne A. Khurana A. Saifi M. A. Bansod S. Anchi P. (2023). Acetaminophen induced hepatotoxicity: an overview of the promising protective effects of natural products and herbal formulations. Phytomedicine 108, 154510. 10.1016/j.phymed.2022.154510 36332383

[B8] Chowdhury A. Nabila J. Adelusi Temitope I. Wang S. (2020). Current etiological comprehension and therapeutic targets of acetaminophen-induced hepatotoxicity. Pharmacol. Res. 161, 105102. 10.1016/j.phrs.2020.105102 32738495

[B9] Conrad M. Sato H. (2012). The oxidative stress-inducible cystine/glutamate antiporter, system x (c) (-): cystine supplier and beyond. Amino Acids 42 (1), 231–246. 10.1007/s00726-011-0867-5 21409388

[B10] Ding Q. Cao F. Lai S. Zhuge H. Chang K. Valencak T. G. (2022). Lactobacillus plantarum ZY08 relieves chronic alcohol-induced hepatic steatosis and liver injury in mice via restoring intestinal flora homeostasis. Food Res. Int. 157, 111259. 10.1016/j.foodres.2022.111259 35761571

[B11] Du K. Ramachandran A. Jaeschke H. (2016). Oxidative stress during acetaminophen hepatotoxicity: sources, pathophysiological role and therapeutic potential. Redox Biol. 10, 148–156. 10.1016/j.redox.2016.10.001 27744120 PMC5065645

[B12] Du Y. C. Lai L. Zhang H. Zhong F. R. Cheng H. L. Qian B. L. (2020). Kaempferol from Penthorum chinense Pursh suppresses HMGB1/TLR4/NF-κB signaling and NLRP3 inflammasome activation in acetaminophen-induced hepatotoxicity. Food Funct. 11 (9), 7925–7934. 10.1039/d0fo00724b 32820776

[B13] Fernandez-Checa J. C. Bagnaninchi P. Ye H. Sancho-Bru P. Falcon-Perez J. M. Royo F. (2021). Advanced preclinical models for evaluation of drug-induced liver injury–consensus statement by the European Drug-Induced Liver Injury Network [PRO-EURO-DILI-NET]. J. Hepatol. 75 (4), 935–959. 10.1016/j.jhep.2021.06.021 34171436

[B14] Forcina G. C. Dixon S. J. (2019). GPX4 at the crossroads of lipid homeostasis and ferroptosis. Proteomics 19 (18), e1800311. 10.1002/pmic.201800311 30888116

[B15] Guimaraes N. S. S. Ramos V. S. Prado-Souza L. F. L. Lopes R. M. Arini G. S. Feitosa L. G. P. (2023). Rosemary (*rosmarinus officinalis* L.) glycolic extract protects liver mitochondria from oxidative damage and prevents acetaminophen-induced hepatotoxicity. Antioxidants (Basel) 12 (3), 628. 10.3390/antiox12030628 36978874 PMC10045355

[B16] Huang M. Wang Y. Wu X. Li W. (2024). Crosstalk between endoplasmic reticulum stress and ferroptosis in liver diseases. Front. Biosci. Landmark Ed. 29 (6), 221. 10.31083/j.fbl2906221 38940044

[B17] Imai H. Matsuoka M. Kumagai T. Sakamoto T. Koumura T. (2017). Lipid peroxidation-dependent cell death regulated by GPx4 and ferroptosis. Curr. Top. Microbiol. Immunol. 403, 143–170. 10.1007/82_2016_508 28204974

[B18] Iuchi K. Takai T. Hisatomi H. (2021). Cell death via lipid peroxidation and protein aggregation diseases. Biology (Basel) 10 (5), 399. 10.3390/biology10050399 34064409 PMC8147787

[B19] Jaeschke H. Adelusi O. B. Akakpo J. Y. Nguyen N. T. Sanchez-Guerrero G. Umbaugh D. S. (2021). Recommendations for the use of the acetaminophen hepatotoxicity model for mechanistic studies and how to avoid common pitfalls. Acta Pharm. Sin. B 11 (12), 3740–3755. 10.1016/j.apsb.2021.09.023 35024303 PMC8727921

[B20] Jehan C. Cartier D. Bucharles C. Anouar Y. Lihrmann I. (2022). Emerging roles of ER-resident selenoproteins in brain physiology and physiopathology. Redox Biol. 55, 102412. 10.1016/j.redox.2022.102412 35917681 PMC9344019

[B21] Jelic M. D. Mandic A. D. Maricic S. M. Srdjenovic B. U. (2021). Oxidative stress and its role in cancer. J. Cancer Res. Ther. 17 (1), 22–28. 10.4103/jcrt.JCRT_862_16 33723127

[B22] Kusama H. Kon K. Ikejima K. Arai K. Aoyama T. Uchiyama A. (2017). Sodium 4-phenylbutyric acid prevents murine acetaminophen hepatotoxicity by minimizing endoplasmic reticulum stress. J. Gastroenterol. 52 (5), 611–622. 10.1007/s00535-016-1256-3 27599972

[B23] Lee Y. J. (2020). The interplay between apoptosis and ferroptosis mediated by ER stress. Apoptosis 25 (11-12), 783. 10.1007/s10495-020-01642-0 33140179 PMC7680372

[B24] Li J. Liu J. Tang Y. Zhang H. Zhang Y. Zha X. (2024). Role of C/EBP homologous protein (CHOP) and Nupr1 interaction in endoplasmic reticulum stress-induced apoptosis of lens epithelial cells. Mol. Biotechnol. 10.1007/s12033-024-01148-z PMC11928426 38771421

[B25] Li L. Wang H. Zhang J. Sha Y. Wu F. Wen S. (2020). SPHK1 deficiency protects mice from acetaminophen-induced ER stress and mitochondrial permeability transition. Cell. Death Differ. 27 (6), 1924–1937. 10.1038/s41418-019-0471-x 31827236 PMC7244772

[B26] Li Y. Deng X. Hu Q. Chen Y. Zhang W. Qin X. (2024). Paeonia lactiflora Pall. ameliorates acetaminophen-induced oxidative stress and apoptosis via inhibiting the PKC-ERK pathway. J. Ethnopharmacol. 329, 118107. 10.1016/j.jep.2024.118107 38599475

[B27] Liao J. Lu Q. Li Z. Li J. Zhao Q. Li J. (2023). Acetaminophen-induced liver injury: molecular mechanism and treatments from natural products. Front. Pharmacol. 14, 1122632. 10.3389/fphar.2023.1122632 37050900 PMC10083499

[B28] Lu Q. Jiang M. H. Jiang J. G. Zhang R. F. Zhang M. W. (2012). Isolation and identification of compounds from Penthorum chinense Pursh with antioxidant and antihepatocarcinoma properties. J. Agric. Food Chem. 60 (44), 11097–11103. 10.1021/jf303755w 23075052

[B29] Luo Y. Lei Y. Zhou H. Chen Y. Liu H. Jiang J. (2024). ARRB1 downregulates acetaminophen-induced hepatoxicity through binding to p-eIF2α to inhibit ER stress signaling. Cell. Biol. Toxicol. 40 (1), 1. 10.1007/s10565-024-09842-z 38252352 PMC10803539

[B30] Luyendyk J. P. Morozova E. Copple B. L. (2024). Good cells go bad: immune dysregulation in the transition from acute liver injury to liver failure after acetaminophen overdose. Drug Metab. Dispos. 52 (8), 722–728. 10.1124/dmd.123.001280 38050055 PMC11257689

[B31] Maiorino M. Conrad M. Ursini F. (2018). GPx4, lipid peroxidation, and cell death: discoveries, rediscoveries, and open issues. Antioxid. Redox Signal 29 (1), 61–74. 10.1089/ars.2017.7115 28462584

[B32] Nishida Xavier da Silva T. Friedmann Angeli J. P. Ingold I. (2022). GPX4: old lessons, new features. Biochem. Soc. Trans. 50 (3), 1205–1213. 10.1042/BST20220682 35758268

[B33] Pei J. Pan X. Wei G. Hua Y. (2023). Research progress of glutathione peroxidase family (GPX) in redoxidation. Front. Pharmacol. 14, 1147414. 10.3389/fphar.2023.1147414 36937839 PMC10017475

[B34] Qin K. Tang H. Ren Y. Yang D. Li Y. Huang W. (2022). Melatonin promotes sirtuin 1 expression and inhibits IRE1α-XBP1S-CHOP to reduce endoplasmic reticulum stress-mediated apoptosis in chondrocytes. Front. Pharmacol. 13, 940629. 10.3389/fphar.2022.940629 36034777 PMC9404507

[B35] Rozpedek W. Pytel D. Mucha B. Leszczynska H. Diehl J. A. Majsterek I. (2016). The role of the PERK/eIF2α/ATF4/CHOP signaling pathway in tumor progression during endoplasmic reticulum stress. Curr. Mol. Med. 16 (6), 533–544. 10.2174/1566524016666160523143937 27211800 PMC5008685

[B36] Shen J. Zhang C. Liu Y. Zhao M. Wang Q. Li P. (2023). L-type calcium ion channel-mediated activation of autophagy in vascular smooth muscle cells via thonningianin A (TA) alleviates vascular calcification in type 2 diabetes mellitus. Eur. J. Pharmacol. 959, 176084. 10.1016/j.ejphar.2023.176084 37806540

[B37] Shu Y. He D. Li W. Wang M. Zhao S. Liu L. (2020). Hepatoprotective effect of *citrus aurantium* L. Against APAP-induced liver injury by regulating liver lipid metabolism and apoptosis. Int. J. Biol. Sci. 16 (5), 752–765. 10.7150/ijbs.40612 32071546 PMC7019131

[B38] Sun X. Wu A. Kwan Law B. Y. Liu C. Zeng W. Ling Qiu A. C. (2020). The active components derived from Penthorum chinensePursh protect against oxidative-stress-induced vascular injury via autophagy induction. Free Radic. Biol. Med. 146, 160–180. 10.1016/j.freeradbiomed.2019.10.417 31689485

[B39] Sun Y. He L. Wang W. Xie Z. Zhang X. Wang P. (2022). Activation of Atg7-dependent autophagy by a novel inhibitor of the Keap1-Nrf2 protein-protein interaction from Penthorum chinense Pursh. attenuates 6-hydroxydopamine-induced ferroptosis in zebrafish and dopaminergic neurons. Food Funct. 13 (14), 7885–7900. 10.1039/d2fo00357k 35776077

[B40] Tan Q. Liu Y. Deng X. Chen J. Tsai P. J. Chen P. H. (2020). Autophagy: a promising process for the treatment of acetaminophen-induced liver injury. Arch. Toxicol. 94 (9), 2925–2938. 10.1007/s00204-020-02780-9 32529281

[B41] Tang S. P. Mao X. L. Chen Y. H. Yan L. L. Ye L. P. Li S. W. (2022). Reactive oxygen species induce fatty liver and ischemia-reperfusion injury by promoting inflammation and cell death. Front. Immunol. 13, 870239. 10.3389/fimmu.2022.870239 35572532 PMC9098816

[B42] Ursini F. Maiorino M. (2020). Lipid peroxidation and ferroptosis: the role of GSH and GPx4. Free Radic. Biol. Med. 152, 175–185. 10.1016/j.freeradbiomed.2020.02.027 32165281

[B43] van der Mijn J. C. Chen Q. Laursen K. B. Khani F. Wang X. Dorsaint P. (2022). Transcriptional and metabolic remodeling in clear cell renal cell carcinoma caused by ATF4 activation and the integrated stress response (ISR). Mol. Carcinog. 61 (9), 851–864. 10.1002/mc.23437 35726553 PMC9378514

[B44] Wan J. Kuang G. Zhang L. Jiang R. Chen Y. He Z. (2020). Hesperetin attenuated acetaminophen-induced hepatotoxicity by inhibiting hepatocyte necrosis and apoptosis, oxidative stress and inflammatory response via upregulation of heme oxygenase-1 expression. Int. Immunopharmacol. 83, 106435. 10.1016/j.intimp.2020.106435 32222641

[B45] Wang H. Li H. Liu Z. Zhu Z. Cao Y. (2024). Activity of thonningianin A against Candida albicans *in vitro* and *in vivo* . Appl. Microbiol. Biotechnol. 108 (1), 96. 10.1007/s00253-023-12996-1 38212967 PMC10784352

[B46] Wang M. Chen Z. Yang L. Ding L. (2021). Sappanone A protects against inflammation, oxidative stress and apoptosis in cerebral ischemia-reperfusion injury by alleviating endoplasmic reticulum stress. Inflammation 44 (3), 934–945. 10.1007/s10753-020-01388-6 33411101

[B47] Wang M. Sun J. Yu T. Wang M. Jin L. Liang S. (2022). Diacerein protects liver against APAP-induced injury via targeting JNK and inhibiting JNK-mediated oxidative stress and apoptosis. Biomed. Pharmacother. 149, 112917. 10.1016/j.biopha.2022.112917 36068777

[B48] Wiseman R. L. Mesgarzadeh J. S. Hendershot L. M. (2022). Reshaping endoplasmic reticulum quality control through the unfolded protein response. Mol. Cell. 82 (8), 1477–1491. 10.1016/j.molcel.2022.03.025 35452616 PMC9038009

[B49] Xie Y. Kang R. Klionsky D. J. Tang D. (2023). GPX4 in cell death, autophagy, and disease. Autophagy 19 (10), 2621–2638. 10.1080/15548627.2023.2218764 37272058 PMC10472888

[B50] Xu J. Zhao L. Zhang X. Ying K. Zhou R. Cai W. (2023). Salidroside ameliorates acetaminophen-induced acute liver injury through the inhibition of endoplasmic reticulum stress-mediated ferroptosis by activating the AMPK/SIRT1 pathway. Ecotoxicol. Environ. Saf. 262, 115331. 10.1016/j.ecoenv.2023.115331 37556956

[B51] Yang Y. He Y. Jin Y. Wu G. Wu Z. (2021). Amino acids in endoplasmic reticulum stress and redox signaling. Adv. Exp. Med. Biol. 1332, 35–49. 10.1007/978-3-030-74180-8_3 34251637

[B52] Yong Y. Yan L. Wei J. Feng C. Yu L. Wu J. (2024). A novel ferroptosis inhibitor, Thonningianin A, improves Alzheimer’s disease by activating GPX4. Theranostics 14 (16), 6161–6184. 10.7150/thno.98172 39431016 PMC11488096

[B53] Yu L. Wang H. Yao Q. Li K. Qu L. Tang B. (2024). Thonningianin A from Penthorum chinense Pursh as a targeted inhibitor of Alzheimer’s disease-related β-amyloid and Tau proteins. Phytother. Res. 38, 4815–4831. 10.1002/ptr.8060 39225174

[B54] Zhang J. Duan D. Song Z. L. Liu T. Hou Y. Fang J. (2021). Small molecules regulating reactive oxygen species homeostasis for cancer therapy. Med. Res. Rev. 41 (1), 342–394. 10.1002/med.21734 32981100

[B55] Zhang J. Guo J. Yang N. Huang Y. Hu T. Rao C. (2022). Endoplasmic reticulum stress-mediated cell death in liver injury. Cell. Death Dis. 13 (12), 1051. 10.1038/s41419-022-05444-x 36535923 PMC9763476

[B56] Zhang S. Zhang S. Bai X. Wang Y. Liu Y. Liu W. (2024). Thonningianin A ameliorated renal interstitial fibrosis in diabetic nephropathy mice by modulating gut microbiota dysbiosis and repressing inflammation. Front. Pharmacol. 15, 1389654. 10.3389/fphar.2024.1389654 39193336 PMC11347433

[B57] Zhang T. T. Yang L. Jiang J. G. (2015). Effects of thonningianin A in natural foods on apoptosis and cell cycle arrest of HepG-2 human hepatocellular carcinoma cells. Food Funct. 6 (8), 2588–2597. 10.1039/c5fo00388a 26119846

[B58] Zhao C. Yu D. He Z. Bao L. Feng L. Chen L. (2021). Endoplasmic reticulum stress-mediated autophagy activation is involved in cadmium-induced ferroptosis of renal tubular epithelial cells. Free Radic. Biol. Med. 175, 236–248. 10.1016/j.freeradbiomed.2021.09.008 34520822

[B59] Zhou X. G. Qiu W. Q. Yu L. Pan R. Teng J. F. Sang Z. P. (2022). Targeting microglial autophagic degradation of the NLRP3 inflammasome for identification of thonningianin A in Alzheimer's disease. Inflamm. Regen. 42 (1), 25. 10.1186/s41232-022-00209-7 35918778 PMC9347127

[B60] Zhou Y. Fan X. Jiao T. Li W. Chen P. Jiang Y. (2021). SIRT6 as a key event linking P53 and NRF2 counteracts APAP-induced hepatotoxicity through inhibiting oxidative stress and promoting hepatocyte proliferation. Acta Pharm. Sin. B 11 (1), 89–99. 10.1016/j.apsb.2020.06.016 33532182 PMC7838028

